# Work outcome in persons with musculoskeletal diseases: comparison with other chronic diseases & the role of musculoskeletal diseases in multimorbidity

**DOI:** 10.1186/s12891-016-1365-4

**Published:** 2017-01-10

**Authors:** Antje van der Zee-Neuen, Polina Putrik, Sofia Ramiro, Andras Keszei, Rob de Bie, Astrid Chorus, Annelies Boonen

**Affiliations:** 1 Internal Medicine/Rheumatology, CAPHRI, Maastricht University Medical Centre, Maastricht University, NL-6202 AZ MAASTRICHT, P/O 5800, Maastricht, Netherlands; 2 Health Promotion, Maastricht University, Maastricht, Netherlands; 3 Rheumatology, Leiden University Medical Center, Leiden, Netherlands; 4 Rheumatology, Hospital Garcia de Orta, Almada, Portugal; 5 Department of Medical Informatics Uniklinik RWTH, Aachen University, Aachen, Germany; 6 Netherlands Organization for Applied Scientific Research, Lifestyle, Leiden, Netherlands

**Keywords:** Living allowance, Multimorbidity, Musculoskeletal diseases, Sick leave, Unemployment, Work disability, Work outcome

## Abstract

**Background:**

Chronic diseases and multimorbidity are increasingly common among persons in working age. This study explores the impact of type, number and combinations of chronic diseases with focus on the role of MSKD on (1) adverse work status (i.e. work disability (WD), economic unemployment (UE) or receiving a living allowance (LA)) and on (2) the occurrence of sick leave.

**Methods:**

Subjects participating in a Dutch household survey, who were ≤65 years and could have paid work, provided data on socio-demographics and nine physician diagnosed chronic diseases. To explore the independent association of each chronic disease, of multimorbidity and of MSKD in context of multimorbidity with 1) work status (employed, WD, LA, UE) and 2) sick leave (SL) in those employed, multinomial logistic regressions and logistic regressions were used, respectively.

**Results:**

Among 5396 subjects, MSKD was the most common morbidity (17%), multimorbidity occurred in 755/5396 (14%), 436/755 (61%) of subjects with multimorbidity had an MSKD. For MSKD the odds of WD, LA and UE were 2.06 [95% CI 1.56;2.71], 2.15[1.18;3.91] and 1.35[0.94;1.96], respectively, compared to being employed and the odds of SL in MSKD were 2.29[1.92;2.73]. Mental diseases had a stronger impact on all these outcomes. The odds for adverse work outcomes increased strongly with an increasing number of diseases. When an MSKD was part of multimorbidity, an additional impact on the association with WD and SL was observed.

**Conclusions:**

Multimorbidity has a stronger impact on all work outcomes compared to single chronic diseases. The presence of the MSKD in the context of multimorbidity amplifies the chance of WD or SL.

**Electronic supplementary material:**

The online version of this article (doi:10.1186/s12891-016-1365-4) contains supplementary material, which is available to authorized users.

## Background

Worldwide, chronic diseases are becoming increasingly prevalent, partly as a consequence of ageing and earlier diagnosis, but also as a consequence of lifestyle changes. According to the Global Burden of Disease initiative, musculoskeletal (MSKD), cardiovascular (CVD), respiratory, mental and gastro-intestinal diseases, diabetes and, somewhat less frequent, cancer are among the most common chronic diseases [1]. In the Netherlands, approximately 31% of the general population reports at least one chronic condition [2]. As the incidence and prevalence of all these conditions is age dependent, the rise is particularly impressive in persons after the age of 50 years. Parallel with this rise, the occurrence of multimorbidity, i.e. the co-occurrence of two or more diseases in one person, is more frequently observed [3, 4].

Existing literature on chronic diseases and multimorbidity mainly focused on the prevalence and the impact on health related quality of life. However, since diagnoses of chronic diseases are increasingly made at younger age, and as in many Western countries the age of retirement is systematically raised to deal with the financial burden associated with the aging of the population, chronic conditions and multimorbidity have potentially large effects on labor force participation [5–8].

Among chronic diseases, MKSD, together with mental diseases, are the main reason of expenditures for work disability (WD) and sick leave (SL) in Western countries when accounting for their prevalence [9, 10]. Nonetheless, comparisons of the relative impact of major chronic diseases on work outcomes have rarely been performed [11, 12]. Yet, such knowledge could be of interest, as similar adverse impact of all chronic diseases on work participation could call for a shared responsibility across medical specialties. Also, the majority of studies concentrate on work disability and/or sick leave as adverse work outcome. However, to obtain a broad view on the consequences of chronic diseases on work participation, it is important to study the impact on sick leave in those still working but also the impact on work disability, (economic) unemployment (UE) and dependence on living allowances (LA). With respect to the latter two outcomes it should be recognized that not all persons with a chronic condition are eligible for an official work disability allowance, but will be vulnerable on the labor market and therefore become (or remain) unemployed and finally dependent on a living allowance.

Existing literature on the impact of multimorbidity on work outcomes is scarce and often restricted to the study of two specific combinations of diseases such as rheumatoid arthritis (RA) and depression or cardiovascular disease [13, 14], and as such addresses the influence of comorbidities on the disease of interest (in this example RA). The lack of interest in multimorbidity is surprising, given that multimorbidity might well be a strong and relevant risk factor for adverse work outcome. Several questions can be raised. First, how strong is the impact of increasing numbers of comorbidities on the different work outcomes. Second, what counts most, the number of morbidities or to specific combinations chronic diseases. Especially from the rheumatologist’s point of view, it would be interesting to understand whether the presence of an MSKD as part of multimorbidity would amplify the impact of multimorbidity on adverse work outcome. An amplifying role of MSKD would argue for special attention for patients that have an MSKD as part of their multimorbidity.

The current study aimed to compare the impact of type, number and combinations of chronic diseases with focus on the role of MSKD on (1) adverse work status (i.e. work disability, economic unemployment or receiving a living allowance) and on (2) the occurrence of sick leave.

We hypothesized that all chronic diseases are associated with an increased risk of work disability compared to the general population and to a lesser extent with unemployment and dependence on a living allowance and that the presence of a MSKD in multimorbidity patterns is associated with a higher risk of adverse work outcome than multimorbidity patterns without MSKD.

## Methods

Data from The National Monitor on Musculoskeletal System 2010, a household survey conducted by the Dutch organization for applied scientific research were used [15]. The study was approved by the Medical Ethical Committee of the Leiden University Medical Center. The detailed approach of sampling and data collection is described elsewhere [16]. In brief, a random sample of Dutch households was contacted by letter and members of the household older than 18 years who were the next to have their birthday were invited to complete and return the questionnaire. Among others, information was collected on socio-demographics (age, gender, highest level of achieved education (i.e. no or primary education, lower professional, middle or secondary professional, general secondary (high school) or higher professional or university education), origin (western, first generation non-western, second generation non-western), lifestyle (weight and height, computed into BMI (kg/m^2^; underweight <18.5; normal: 18.5- < 25.0; pre-obese: 25.0- < 30.0; obese: ≥30.0)); smoking-status (never, past, current)), nine self-reported physician-diagnosed diseases over the last year (i.e. MSKD (defined as severe, persisting complaints in or around joints), diabetes, cardiovascular disease (CVD, defined as hypertension, cerebral hemorrhage/stroke, myocardial infarction, other heart-diseases or peripheral vascular disease), cancer, migraine, respiratory disease, skin disease, mental diseases and gastrointestinal disease), work status (being employed, being retired, being economically unemployed, WD, receiving LA, being housewife or houseman, being a student) and having been on sick leave (SL (yes/no)) during the past 12 months when employed. In this study, we considered ≥ 2 chronic diseases in one person as multimorbidity [3, 4]. The presence of one of the nine chronic diseases, regardless of the presence of other diseases, was referred to as the ‘index disease’ and the presence of one of the nine diseases only (no other disease present) was referred to as ‘single disease’.

### Statistical analyses

Persons of working age (18–65 years) who had paid work, were WD, UE or depending on a LA were included in the analyses when work status was the outcome. When SL in the last 12 months was the outcome, the sample was restricted to those with paid work who answered the question on sick leave.

### Work disability, economic unemployment and receiving a living allowance

To assess the odds to be WD, UE or depending on a LA for subjects with the different morbidities or morbidity patterns compared to having paid work, multinomial regressions were used. Multinomial regressions are useful when the outcome (having no work) can be further classified in different categories (work disabled, economically unemployed or receiving a living allowance).

First, a multinomial regression was performed with the different chronic diseases (index disease compared to no index disease) as the independent variables of interest. Next, a multinomial regression was performed with the number of morbidities (1; 2; 3; ≥4 diseases, reference = no disease reported) as independent variable of interest. Thirdly, a multinomial regression was computed with single diseases (i.e. presence of only one disease) and multimorbidity including MSKD and multimorbidity excluding MSKD as independent variables of interest (reference = no disease). In a scenario analysis, this model was repeated when those with multimorbidity were further classified into 2 or ≥3 diseases with or without MSKD. From the results of the model with the number of morbidities, the predicted probability of work disability with increasing morbidities and age was calculated for a non-smoking subject with middle or professional secondary education and normal BMI, averaged for gender.

### Sick leave

To explore the association of the different chronic diseases (index disease), the number of diseases, or single diseases and multimorbidity in- and excluding MSKD with sick leave logistic regressions were computed. In a scenario analysis, the model including single diseases and multimorbidity was repeated when those with multimorbidity were further classified into 2 or ≥3 diseases with or without MSKD.

### Model development

In all models, age, gender, BMI, smoking status, origin and education were considered potential confounders. Manual forward selection was used to develop fully adjusted models. Variables remained in the multivariable model when they were either significantly associated with the outcome (*p* ≤ 0.05) or confounded the association of the disease (dependent) with the outcome (i.e. ≥10% change in the coefficient). All analyses were performed on complete cases using STATA statistical software 12.0 [17].

## Results

The questionnaire was returned by 8904 (22.4%) subjects of whom 5396 were ≤65 years and were either employed (*n* = 4805; 89%), WD (*n* = 350; 7%), depending on LA (*n* = 57; 3%) or UE (*n* = 184; 3%).

The mean age was 46 years (SD 11) and 3008 (56%) were women (Table 1). MSKD was reported by 925 subjects (17%), 662 (12%) had CVD, 180 (3%) diabetes, 96 (2%) cancer, 355 (7%) respiratory disease, 359 (7%) skin disease, 376 (7%) mental disease, 164 (3%) a bowel (i.e. gastro-intestinal) disease and 275 (5%) migraine. Multimorbidity was present in 14% (*n* = 755) of the cases. Of all persons with 2 morbidities 265/490 (54%) reported an MSKD and of all persons with ≥3 morbidities 198/265 (75%) reported an MSKD (Table 2).

**Table 1:** Characteristics of the study population* (*N* = 5396)

Age, mean (SD)	45.8 (11.0)
Women, *n* (%)	3008 (55.8)
Work status	
Paid work	4,805 (89.1)
Work disabled	350 (6.5)
Unemployed	184 (3.4)
Living allowance	57 (1.1)
BMI, *n* (%)	
Underweight	69 (1.3)
Normal	2853 (52.9)
Pre-obese	1784 (33.1)
Obese	690 (12.8)
Level of education, *n* (%)	*n* = 5357
Primary school or no education	105 (2.0)
Lower professional school	544 (10.2)
Middle or professional secondary school	1677 (31.3)
Secondary education	567 (10.6)
University education	2464 (46.0)
Smoking status, *n* (%)	*n* = 5381
Current smoker	1057 (19.6)
Never smoked	2535 (47.1)
Former smoker	1789 (23.3)
Number of morbidities^a^, *n* (%)	
0	3156 (58.5)
1	1485 (27.5)
2	490 (9.1)
≥ 3	265 (4.9)
Sick leave during past 12 months in subsample with paid work	*n* = 4805
Yes, sick leave n (%)	2264 (47.1)

^a^Population is ≤ 65 years, has either paid work, is work disabled, unemployed or is depending on a living allowance)

**Table 2:** Characteristics of study population (paid work, work disabled, unemployed, living allowance, aged ≤ 65 years) by index diseases

	Musculoskeletal disease(*n* = 925 (17.1%))	Cardiovascular disease(*n* = 662 (12.3%))	Diabetes(*n* = 180 (3.3%))	Cancer(*n* = 96 (1.8%))	Respiratory disease(*n* = 355 (6.6%))	Skin disease(*n* = 359 (6.7%))	Mental disease(*n* = 376 (7.0%))	Bowel disease(*n* = 164 (3.0%))	Migraine(*n* = 275 (5.1%))	No chronic disease(*n* = 3,156 (58.5%))
Age, mean (SD)	49.0 (10.5)	53.3 (8.8)	54.8 (8.0)	54.0 (8.5)	48.3 (11.0)	46.7 (11.0)	46.0 (11.0)	46.6 (11.1)	45.2 (10.9)	44.0 (10.8)
Women, n (%)	563 (60.9)	333 (50.3)	72 (40.0)	63.0 (65.6)	227 (63.9)	208 (57.9)	253 (58.0)	124 (75.6)	231 (84.0)	1685 (53.4)
Work status										
Paid work	706 (76.3)	509 (76.9)	115 (63.9)	64 (66.7)	265 (74.7)	281 (78.3)	216 (57.5)	108 (65.9)	203 (73.8)	2998 (95.0)
Work disabled	152 (16.4)	113 (17.1)	46 (25.6)	26 (27.1)	66 (18.6)	56 (15.6)	121 (32.2)	46 (28.1)	45 (16.4)	86 (2.7)
Unemployed	43 (4.7)	25 (3.8)	11 (6.1)	5 (5.2)	13 (3.7)	17 (4.7)	25 (6.7)	6 (3.7)	23 (8.4)	56 (1.8)
Living allowance	24 (2.6)	15 (2.3)	8 (4.4)	1 (1.0)	11 (3.1)	5 (1.4)	14 (3.7)	4 (2.4)	4 (1.5)	16 (0.5)
BMI, *n* (%)									*n* = 269	
Underweight	15 (1.6)	2 (0.3)	1 (0.6)	-	4 (1.1)	6 (1.7)	11 (2.9)	4 (2.4)	7 (2.6)	42 (1.3)
Normal	387 (41.8)	200 (30.2)	34 (18.9)	37 (38.5)	143 (40.3)	173 (48.2)	179 (47.6)	79 (48.2)	145 (52.7)	1858 (58.9)
Pre-obese	340 (36.8)	261 (39.4)	66 (36.7)	39 (40.6)	121 (34.1)	122 (34.0)	108 (28.7)	57 (34.8)	76 (27.6)	980 (31.1)
Obese	183 (19.8)	199 (30.1)	79 (43.9)	20 (20.8)	87 (24.5)	58 (16.2)	78 (20.8)	24 (14.6)	47 (17.5)	225 (7.1)
Level of education, *n* (%)	*n* = 922	*n* = 656	*n* = 179		*n* = 352	*n* = 358	*n* = 374		*n* = 274	*n* = 3133
Primary school or no education	29 (3.2)	25 (3.8)	11 (6.2)	4 (4.2)	13 (3.7)	12 (3.4)	9 (2.4)	7 (4.3)	3 (1.1)	43 (1.3)
Lower professional school	154 (16.7)	102 (15.6)	37 (20.7)	15 (15.6)	59 (16.8)	48 (13.4)	45 (12.0)	22 (13.4)	38 (13.9)	244 (7.8)
Middle or professional secondary school	331 (35.9)	245 (37.4)	57 (31.8)	28 (29.2)	121 (34.4)	118 (33.0)	126 (33.7)	56 (34.2)	94 (34.2)	920 (29.4)
Secondary education	90 (9.7)	75 (11.4)	20 (11.2)	21 (21.9)	32 (10.5)	40 (11.2)	52 (13.9)	18 (11.0)	34 (12.4)	311 (9.9)
University education	318 (34.5)	209 (31.9)	54 (30.2)	28 (29.2)	127 (36.1)	140 (39.1)	142 (38.0)	61 (37.2)	105 (38.3)	1615 (51.5)
Smoking status, *n* (%)	*n* = 924	*n* = 660	*n* = 179		*n* = 353			*n* = 163		*n* = 3145
Current smoker	217 (23.5)	131 (19.9)	42 (23.5)	20 (20.8)	73 (20.7)	74 (20.6)	118 (31.4)	41 (25.2)	64 (23.3)	567 (18.0)
Never smoked	364 (39.4)	248 (37.6)	50 (27.9)	35 (36.5)	155 (43.9)	141 (39.3)	122 (32.5)	77 (47.2)	128 (46.6)	1610 (51.2)
Former smoker	343 (37.1)	281 (42.6)	87 (48.6)	41 (42.7)	125 (35.4)	144 (40.1)	136 (36.2)	45 (27.6)	83 (30.1)	968 (30.8)
Number of co- morbidities, *n* (%)										
0	462 (50.0)	289 (43.7)	46 (25.6)	39 (40.6)	143 (40.3)	175 (48.8)	162 (43.1)	65 (39.6)	123 (44.7)	3156 (100.0)
1	265 (28.7)	197 (29.8)	62 (34.4)	23 (24.0)	103 (29.0)	101 (28.1)	107 (28.5)	49 (29.9)	79 (28.7)	N/A
2	132 (14.3)	106 (16.0)	40 (22.2)	21 (21.9)	61 (17.2)	58 (16.2)	67 (17.8)	24 (14.6)	50 (18.2)	N/A
≥ 3	66 (7.1)	70 (10.6)	32 (17.8)	13 (13.5)	48 (13.5)	25 (6.9)	40 (10.6)	26 (15.9)	23 (8.4)	N/A
Sick leave during past 12 months in subsample with paid work										
	*n* = 706	*n* = 509	*n* = 115	*n* = 64	*n* = 265	*n* = 281	*n* = 216	*n* = 108	*n* = 203	*n* = 2992
Yes, sick leave *n* (%)	455 (64.5)	257 (50.5)	66 (57.4)	40 (62.5)	154 (58.1)	154 (54.8)	164 (75.9)	69 (63.9)	136 (67.0)	1173 (39.2)

In the total group, the disease that was most prevalent among persons with adverse work outcome was MSKD: 43% (152/350) of those with a WD, 42% (24/57) of those with a LA and 23% (43/184) of those with UE had an MSKD.

An overview of the characteristics of the subsample with paid work (*n* = 4805), in which the occurrence of sick leave in the past year was studied, is presented in Additional file 1. MSKD was reported by 706 (15%), multimorbidity was present in 493 (10%) subjects and of those with multimorbidity 286 (58%) reported an MSKD.

### Work disability, economic unemployment or receiving a living allowance compared to paid work

The model addressing the impact of the different chronic disease on work status showed that patients with MSKD had significantly higher odds to be WD (OR 2.06 [95% CI 1.56; 2.71]) and have a LA (2.15 [1.18; 3.91]) but not to be UE (1.35 [0.94; 1.96]) when using “paid work” as the reference outcome. Similar patterns were observed for other diseases. However, the strongest association with all work outcomes was seen for mental diseases (Table 3).

**Table 3:** Adjusted association between the presence of a chronic disease (index disease) and work disability, dependence on living allowances, and economic unemployment compared to ‘being employed’

Index diseases^b^	Work disabled^a^ (OR [95% CI])	Living allowances^a^ (OR [95% CI])	Unemployed^a^ (OR [95% CI])
Musculoskeletal disease	2.06 [1.56; 2.71]	2.15 [1.18; 3.91]	1.35 [0.94; 1.96]
Cardiovascular disease	1.61 [1.18; 2.21]	1.29 [0.64; 2.62]	0.88 [0.55; 1.40]
Diabetes	2.32 [1.47; 3.67]	3.40 [1.38; 8.37]	1.88 [0.95; 3.72]
Cancer	3.26 [1.82; 5.85]	0.69 [0.09; 5.49]	1.73 [0.67; 4.48]
Respiratory disease	1.72 [1.16; 2.55]	2.02 [0.94; 4.38]	1.08 [0.59; 1.94]
Skin disease	1.96 [1.34; 2.86]	1.02 [0.38; 2.75]	1.37 [0.81; 2.32]
Mental disease	10.54 [7.69; 14.44]	5.60 [2.77; 11.34]	3.03 [1.91; 4.80]
Bowel disease	2.89 [1.74; 4.79]	1.65 [0.52; 5.29]	1.10 [0.46; 2.60]
Migraine	2.03 [1.30; 3.18]	1.13 [0.38; 3.40]	3.10 [1.90; 5.04]

Results of multivariable multinomial regression model adjusted for age, gender, education, BMI, smoking status (*n* = 5340)

Abbreviations: *OR* odds ratio, *CI* confidence interval

^a^Paid work is the reference outcome

^b^Absence of index disease is the reference category

**Fig. 1:**
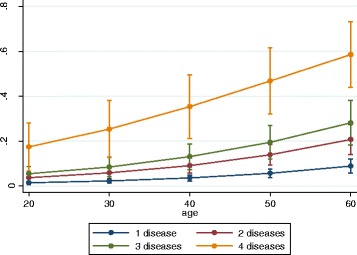
Predicted probability* of work disability by number of morbidities at indicated age. *Predicted probability for non-smoking subjects with middle or professional secondary education and normal BMI based on model estimates shown in Table 3

In the model addressing the association of an increasing number of morbidities with work status, the odds of WD in persons with one morbidity were OR 3.31 [2.35; 4.65]. Each additional morbidity was associated with a steep increase in odds of WD (Table 4). Figure 1 illustrates the predicted probability of WD with an increasing number of morbidities and with increasing age. The odds of depending on *LA or being UE* became significantly increased when more than one morbidity was present

**Table 4:** Adjusted association between the number of diseases and multimorbidity, in- and excluding musculoskeletal disease, and work status compared to ‘being employed’

Number of diseases^Ϯ^	Work disabled* OR [95% CI]	Living allowances* OR [95% CI]	Unemployed* OR [95% CI]
1	3.31 [2.35;4.65]^a^	1.46 [0.69; 3.10]	1.32 [0.92;1.88]
2	9.16 [6.32;13.27]^a^	3.76 [1.67; 8.46]^a^	2.55 [1.63;4.00]^a^
3	14.03 [8.88; 22.16]	6.37 [2.46; 16.51]	3.08 [1.60;5.92]
≥ 4	30.33 [14.87; 61.85]^a^	16.29 [4.94; 53.67]	4.50 [1.58;13.62]
Single and multimorbidity in- and excluding MSKD^Ϯ^			
Single morbidity being an MSKD	2.00 [1.19; 3.39]^b^	1.39 [0.49; 3.99]	1.23 [0.71; 2.14]
Single morbidity other than MSKD	3.91 [2.73; 5.60]	1.48 [0.64; 3.47]	1.36 [0.91; 2.02]
Multimorbidity including MSKD	14.35 [10.02; 20.56]	6.32 [2.99; 13.35]	2.84 [1.78; 4.53]
Multimorbidity excluding MSKD	9.92 [6.54; 15.05]	3.34 [1.25; 8.94]	2.65 [1.54; 4.58]

Results of multivariable multinomial regression model adjusted for age, gender, education, BMI & smoking-status (*n* = 5340)

Abbreviations*: MSKD* musculoskeletal disorder*, OR* odds ratio, *CI* confidence interval

*Paid work is the reference outcome

ϮNo morbidity is the reference category

^a^Significantly different from estimate for previous disease count (i.e.*1* vs. *0; 2* vs. *1 etcetera)*

^b^Significantly different from estimate for single morbidity other than MSKD

^c^Significantly different from estimate for multimorbidity excluding MSKD

The model addressing the association of single diseases or multimorbidity with or without MSKD with work status revealed that when an MSKD was part of multimorbidity, the odds of WD, dependence on LA and UE increased (although not significantly) (Table 4). When exploring the influence of the presence of an MSKD in the context of 2 or ≥3 morbidities similar trends (adverse influence of presence of an MSKD) were seen (Additional file 2). Additional analyses on the impact of specific combinations of MSKD with each of the other chronic disease revealed that the co-occurrence of MSKD, cancer and mental diseases had the largest impact on WD and that co-occurrence of MSKD and mental diseases had the strongest association with dependence on LA and UE (Additional file 3).

### Sick leave

Among those employed, persons with MSKD had increased odds for sick leave (2.29 [1.92; 2.73]). Persons with mental diseases (3.24 [2.33;4.52]) or cancer (2.20 [1.28;3.76]) had higher or comparable odds, respectively (Table 5).

**Table 5:** Odds of sick leave in the past 12 months by morbidity (types and number)

	OR [95% CI]
Index diseases^+^	
Musculoskeletal disease	2.29 [1.92; 2.73]
Cardiovascular disease	1.21 [0.99; 1.49]
Diabetes	1.46 [0.97; 2.20]
Respiratory disease	1.37 [1.05; 1.79]
Mental disease	3.24 [2.33; 4.52]
Cancer	2.20 [1.28; 3.76]
Skin disease	1.24 [0.96; 1.61]
Bowel disease	1.54 [1.01; 2.34]
Migraine	1.91 [1.39; 2.63]
Number of morbidities*	
1	2.00 [1.75; 2.30]^a^
2	2.72 [2.14; 3.44]^a^
3	4.22 [2.74; 6.51]
≥ 4	8.19 [2.73; 24.59]
Single morbidity/multimorbidity in- and excluding MSKD*	
Musculoskeletal disease	2.50 [2.01; 3.10]^b^
Any disease excl. MSKD	1.81 [1.55; 2.12]
Multimorbidity including MSKD	4.04 [3.07; 5.34]^c^
Multimorbidity excluding MSKD	2.40 [1.78; 3.24]

Results of multivariable logistic regression models adjusted for age, gender, level of education, origin, BMI and smoking status *(n =* 4770*)*

Abbreviations*: MSKD* musculoskeletal disorder*, OR* odds ratio*, CI* confidence interval

^*+*^reference category = population without the index disease

*reference category = healthy population

^a^Significantly different from estimate for previous disease count *(i.e.1* vs*. 0; 2* vs*. 1 etcetera)*

^b^Significantly different from estimate for single morbidity other than MSKD

^c^Significantly different from estimate for multimorbidity excluding MSKD

While the odds of sick leave were significantly higher in persons having (any) single morbidity compared to those without a disease (OR 2.00 [95% CI 1.75; 2.30]), the odds of sick leave increased with increasing number of morbidities (Table 5). The odds of sick leave were significantly higher when a MSKD was part of the multimorbidity (OR 1.70 [95% CI 1.15;2.51]) (Table 5). An amplifying effect of the presence of an MSKD on SL was seen irrespective of the number of morbidities present (Additional file 4).

## Discussion

Although we hypothesized that MSKD would have stronger associations with work outcomes when compared to other conditions, data revealed that all chronic diseases were associated with WD and depending on LA. On the same line, the majority of conditions were also associated with the likelihood of incurring SL in the previous 12 months. As expected, when the number of morbidities increases, the odds of any adverse work outcome increased as well. Consistent with our hypothesis, the presence of MSKD in the context of multimorbidity tended to amplify the risk for adverse work outcome and especially of sick leave.

While from a societal point of view MSKD and mental diseases account for the majority of expenditures for SL and WD in most of Western countries, our data emphasize that for MSKD this is mainly attributable to their higher prevalence among persons in the working age range. When comparing the *relative impact* on adverse work outcomes across diseases, mental diseases clearly have the largest impact on WD and LA, but for all other chronic diseases the association with WD and depending on a LA was in the same range. An exception was the association with sick leave, where no impact for CVD, diabetes and skin diseases was observed, but where patients with mental disease followed by MSKD and cancer incurred substantial SL in the previous 12 months.

Only few studies compared the relative impact of distinct chronic diseases on the different types of work outcomes. In an international study across 10 European countries including 11,462 patients, Alavinia et al. compared the association of heart attack, hypertension, stroke, diabetes, chronic lung disease and asthma and depressive symptoms with economic unemployment, after excluding persons with official work disability [12]. They found that the chronic diseases studied had no impact on economic UE, except for the presence of depressive symptoms. On the other hand, a prospective study among 707 Finnish construction workers showed that health problems predict long term unemployment. In this study unemployment was defined as joblessness longer than 24 months and it is unclear to what extent this was related to economic unemployment or official work disability [18]. However, our data suggest that not all persons with a chronic condition are eligible for an official work disability allowance, but apparently they are still vulnerable on the labor market and therefore become (or remain) unemployed and finally become dependent on a living allowance.

The large impact of MSKD on increased sick leave, especially in those with mental disease and MSKD, has been previously identified [19, 20], but our study showed that mental diseases and cancer had an equally large or even larger impact. On the same line, Kessler et al. showed (although not distinguishing between paid and unpaid work) that when comparing the impact of chronic diseases (i.e. arthritis, asthma, diabetes, high blood pressure, autoimmune disease, ulcers, heart disease and several mental diseases) on the number of days that work had to be cut back or in which participants were unable to work, mental diseases had the strongest impact on reduced work-days followed by ulcers and arthritis [11]. Although our study concentrated on sickness absence in those with paid work, it is not surprising that the same diseases also provided problems in being productive in unpaid work.

Multimorbidity had a consistent and strong impact on all aspects of work outcome, also consistently on sick leave and economic UE. This suggests that the combination of individual chronic diseases is additionally hindering work opportunities, which is supported by the steep increase in odds of unfavorable work outcome with increasing number of diseases. Of interest, in the context of multimorbidity, the presence of an MSKD tended to amplify the adverse impact on work outcome, although only statistically significant when sick leave was the outcome. Unfortunately, our sample was not large enough to retrieve stable estimates for the association of all combinations of two diseases. Within this limitation, analysis revealed that the combination of MSKD and mental diseases as well as also cancer and MSKD had indeed the largest impact on adverse work outcome. The detrimental combination of depression and RA on WD has been shown previously [14, 21]. However, to our knowledge no further literature exists regarding the impact of multimorbidity in- and excluding an MSKD on adverse work outcome.

Some limitations deserve attention. The overall response rate of 22.4% was low but comparison of socio-demographic characteristics with data of the central office for statistics in the Netherlands only showed minor differences (Additional file 5), which suggests our results are fairly generalizable. Further, data on chronic diseases and the outcomes were self-reported which might result in misclassification of exposure and outcome. However, studies suggest that self-reported diagnoses that specify that the disease should be confirmed by a physician (which was the case in this study) reduce the bias [22, 23]. While more specific data on diagnoses included in MSKD was available and analyses in subgroups would have been favorable, we were not able to analyze the specific diagnoses in MSKD group due to small numbers of subjects of working age in each diagnosis group. As in all cross-sectional studies, our findings can only be interpreted in terms of associations and no conclusions on causality can be drawn. Furthermore, our sample concerns the Dutch population only and therefore may have limited generalizability to other countries. Existing difference in criteria to receive a work disability pensions, or a LA, and in prevailing UE rates, will obviously impact the likelihood of being eligible for a WD or the likelihood to become long-term UE or dependent on a LA. Last but not least, we have no data on specific diagnoses within the larger disease groups, nor on objective severity and duration of disease, hampering detailed information on disease characteristics related to adverse work outcome.

The strength of this study is the variety of work outcomes included and the novel insights it gives into the impact of (multi)morbidity on these outcomes. Our results call for common action for all those involved in care for patients with chronic diseases to address work ability and assess risk for adverse work outcome in all chronic diseases and especially in those with multimorbidity. First and foremost, prevention of adverse work outcome is mandatory and a duty for all professionals involved in the care of persons with chronic diseases. It is known that sick leave is a strong predictor of WD, and especial persons who are already on sick leave should receive additional support from the medical team to remain in labor force. Rheumatologists may play a role in the identification of patients at risk for adverse work outcome and support them in gaining self-management skills that help in maintaining their work. Further, in currently economically unfavorable times, efforts should be made by policy makers and employers to prevent that persons with (multiple) chronic disease will lose their job and/or to help them return to (other) work.

## Conclusion

In conclusion, chronic diseases (the presence of any of them) and specifically multimorbidity have an important association with the broad spectrum of adverse work outcomes. Our findings emphasize the need to develop common strategies among all chronic disease to improve work outcome in daily clinical care. Professionals in rheumatological care could take the lead for such initiatives.

## Additional files

Additional file 1: Characteristics of study population with paid work ≤ 65 years (DOCX 14 kb)
Additional file 2: Association of multimorbidity of 2 or ≥3 diseases in- and excluding musculoskeletal disease with work status compared to ‘being employed’, comparison of odds of work disability, dependence on living allowances and unemployment between multimorbidity patterns including a musculoskeletal disease and multimorbidity patterns without a musculoskeletal disease. (DOCX 14 kb)
Additional file 3: Association of specific disease combinations in- and excluding musculoskeletal disease with work status compared to ‘being employed’, odds of work disability, dependence on living allowances and unemployment for musculoskeletal disease, cardiovascular disease, diabetes, respiratory disease, mental disease, cancer, skin disease, bowel disease, migraine, combinations of the latter eight diseases with musculoskeletal disease, any two diseases other than musculoskeletal and combinations of 3 diseases with and without MSKD. (DOCX 16 kb)
Additional file 4: Association of multimorbidity of 2 or ≥3 diseases in- and excluding musculoskeletal disease with sick leave in the past 12 months in employed population ≤65 years, comparison of odds of sick leave between multimorbidity patterns including a musculoskeletal disease and multimorbidity patterns without a musculoskeletal disease. (DOCX 14 kb)
Additional file 5: Characteristics of the study sample and general Dutch population (DOCX 16 kb)

## Abbreviations

CVD: Cardiovascular
LA: Living allowance
MSKD: Musculoskeletal diseases
SL: Sick leave
UE: Unemployed
WD: Work disabled
