# Supplementary material for: Work outcome in persons with musculoskeletal diseases: comparison with other chronic diseases & the role of musculoskeletal diseases in multimorbidity
Source: BMC Musculoskelet Disord. 2017 Jan 10;18:10. doi: 10.1186/s12891-016-1365-4 (PMC5223391; doi:10.1186/s12891-016-1365-4)
Supplement: Additional file 1: — Characteristics of study population with paid work ≤ 65 years (DOCX 14 kb) [file 12891_2016_1365_MOESM1_ESM.docx]

| **Additional file 1 Characteristics of study population with paid work ≤ 65 years** n=4,805 | |
| --- | --- |
| Age, mean (SD) |  |
| Women, n (%) | 2669 (55.6) |
| **BMI, n (%)** |  |
| underweight | 57 (1.2) |
| normal | 2615 (54.4) |
| pre-obese | 1598 (33.3) |
| obese | 535 (11.1) |
| **Level of education, n (%)** | n=4,770 |
| Primary school or no education | 55 (1.2) |
| Lower professional school | 421 (8.8) |
| Middle or professional secondary school | 1467 (30.8) |
| Secondary education | 489 (10.3) |
| University education | 2338 (49.0) |
| **Smoking status, n (%)** | n=4,791 |
| Current smoker | 858 (17.9) |
| Never smoked | 2361 (49.3) |
| Former smoker | 1572 (32.8) |
| **Number of morbidities, n (%)** |  |
| 0 | 2998 (62.4) |
| 1 | 1314 (27.4) |
| 2 | 358 (7.45) |
| ≥3 | 135 (2.8) |
| **Sick leave during past 12 months** |  |
| Yes, sick leave n (%) | 2264 (47.1) |
| **Index-diseases** |  |
| Musculoskeletal disease | 706 (14.7) |
| Cardiovascular disease | 509 (10.6) |
| Diabetes | 115 (2.4) |
| Cancer | 64 (1.3) |
| Respiratory disease | 265 (5.5) |
| Skin disease | 281 (5.9) |
| Mental disease | 216 (4.5) |
| Bowel disease | 108 (2.3) |
| Migraine | 203 (4.2) |
