# Supplementary material for: Work outcome in persons with musculoskeletal diseases: comparison with other chronic diseases & the role of musculoskeletal diseases in multimorbidity
Source: BMC Musculoskelet Disord. 2017 Jan 10;18:10. doi: 10.1186/s12891-016-1365-4 (PMC5223391; doi:10.1186/s12891-016-1365-4)
Supplement: Additional file 2: — Association of multimorbidity of 2 or ≥3 diseases in- and excluding musculoskeletal disease with work status compared to ‘being employed’, comparison of odds of work disability, dependence on living allowances and unemployment between multimorbidity patterns including a musculoskeletal disease and multimorbidity patterns without a musculoskeletal disease. (DOCX 14 kb) [file 12891_2016_1365_MOESM2_ESM.docx]

| **Additional file 2**  **Association of multimorbidity of 2 or ≥3 diseases in- and excluding musculoskeletal disease with work status compared to ‘being employed’** | | | |
| --- | --- | --- | --- |
| **Single morbidity/**  **Multimorbidity in- and excluding MSKD^Ϯ^** | **Work disabled***  **OR [95% CI]** | **Living allowances***  **OR [95% CI]** | **Unemployed***  **OR [95% CI]** |
| Musculoskeletal disease | 2.00 (1.19; 3.39)^a^ | 1.39 (0.49; 3.99) | 1.23 (0.71; 2.14) |
| Any disease excl. MSKD | 3.91 (2.73; 5.60) | 1.48 (0.64; 3.47) | 1.36 (0.91; 2.02) |
| 2 diseases incl. MSKD | 9.22 (5.98; 14.23) | 5.61 (2.32; 13.55) | 2.66 (1.51; 4.68) |
| 2 diseases excl. MSKD | 8.84 (5.59; 14.00) | 1.74 (0.47; 6.54) | 2.43 (1.31; 4.51) |
| ≥3 diseases incl. MSKD | 23.73 (15.42; 36.53) | 7.70 (3.05; 19.45) | 3.21 (1.62; 6.35) |
| ≥3 diseases excl. MSKD | 14.89 (7.73; 28.71) | 10.45 (2.86; 38.13) | 3.58 (1.34; 9.54) |
| *Results of multivariable multinomial regression model adjusted for age, gender, education, BMI & smoking-status, n=5,340*  ** Paid work is reference outcome*  *Ϯ No morbidity is reference category*  *^a^ Significantly different from estimate for previous disease count (i.e.1 vs. 0; 2 vs. 1 etcetera)*  *^b^ Significantly different from estimate for single morbidity other than MSKD*  *^c^ Significantly different from estimate for multimorbidity excluding MSKD  Abbreviations: MSKD: musculoskeletal disorder; OR: odds ratio; CI: confidence interval* | | | |
