# Supplementary material for: Work outcome in persons with musculoskeletal diseases: comparison with other chronic diseases & the role of musculoskeletal diseases in multimorbidity
Source: BMC Musculoskelet Disord. 2017 Jan 10;18:10. doi: 10.1186/s12891-016-1365-4 (PMC5223391; doi:10.1186/s12891-016-1365-4)
Supplement: Additional file 3: — Association of specific disease combinations in- and excluding musculoskeletal disease with work status compared to ‘being employed’, odds of work disability, dependence on living allowances and unemployment for musculoskeletal disease, cardiovascular disease, diabetes, respiratory disease, mental disease, cancer, skin disease, bowel disease, migraine, combinations of the latter eight diseases with musculoskeletal disease, any two diseases other than musculoskeletal and combinations of 3 diseases with and without MSKD. (DOCX 16 kb) [file 12891_2016_1365_MOESM3_ESM.docx]

| **Additional file** 3  **Association of specific disease combinations in- and excluding musculoskeletal disease with work status compared to ‘being employed’; OR [95% CI]** | | | |
| --- | --- | --- | --- |
| **Single morbidity/**  **Multimorbidity in- and excluding MSKD^Ϯ^** | **Work disabled*** | **Living allowances*** | **Unemployed*** |
| Musculoskeletal disease | 1.89 [1.12;3.19] | 1.38 [0.48;3.95] | 1.24 [0.71;2.14] |
| Cardiovascular disease | 1.22 [0.62; 2.40] | 1.28 [0.35;4.69] | 0.89 [0.42;1.89] |
| Diabetes | 2.91 [1.04;8.10] | 2.05 [0.24;17.68] | 2.02 [0.60;6.82] |
| Respiratory disease | 2.61 [1.14;5.97] | -^1^ | 1.09 [0.39;3.02] |
| Mental disease | 21.11 [13.10;34.03] | 3.90 [0.85;17.91] | 2.21 [0.99;4.92] |
| Cancer | 4.03 [1.43;11.37] | -^1^ | 0.95 [0.13;7.08] |
| Skin disease | 2.33 [1.10;4.92] | 0.83 [0.11;6.48] | 1.57 [0.74;3.31] |
| Bowel disease | 1.44 [0.33;6.26] | 2.24 [0.25;20.18] | 1.05 [0.25;4.43] |
| Migraine | 1.79 [0.63;5.14] | 2.59 [0.53;12.73] | 2.63 [1.22;5.64] |
| MSKD & Diabetes | 13.56 [4.06; 45.25] | 13.24 [1.27; 138.09] | 2.78 [0.34; 22.76] |
| MSKD & CVD | 4.52 [2.03; 10.04] | 1.56 [0.17; 14.53] | 0.54 [0.07; 4.02] |
| MSKD & Cancer | 35.49 [5.57; 226.13] | -^1^ | -^1^ |
| MSKD & Respiratory disease | 5.21 [1.84; 14.78] | 3.34 [0.38; 29.05] | 1.07 [0.14; 8.04] |
| MSKD & Skin disease | 4.68 [1.90; 11.54] | -^1^ | 1.24 [0.27; 5.60] |
| MSKD & Mental disease | 30.81 [13.93; 68.12] | 23.29 [6.18; 87.75] | 7.95 [2.86; 22.10] |
| MSKD & Migraine | 4.89 [1.61; 14.91] | 5.36 [0.60; 48.10] | 6.38 [2.30; 17.65] |
| MSKD & Bowel disease | 3.99 [1.09; 14.59] | 7.66 [1.19; 49.51] | 0.94 [0.11; 8.01] |
| Any 2 diseases excluding MSKD | 8.23 [5.21; 13.01] | 1.75 [0.47; 6.55] | 2.42 [1.31; 4.49] |
| ≥3 diseases incl. MSKD | 23.73 (15.42; 36.53) | 7.70 (3.05; 19.45) | 3.21 (1.62; 6.35) |
| ≥3 diseases excl. MSKD | 14.89 (7.73; 28.71) | 10.45 (2.86; 38.13) | 3.58 (1.34; 9.54) |
| *Results of multivariable multinomial regression model with age, gender, education, BMI & smoking-status, single diseases and combinations of ≥ diseases as covariates (n=5340)*  ** Paid work is reference outcome*  *Ϯ No morbidity is reference category*  *^1^ not estimated due to insufficient sample size* | | | |
