# Supplementary material for: Work outcome in persons with musculoskeletal diseases: comparison with other chronic diseases & the role of musculoskeletal diseases in multimorbidity
Source: BMC Musculoskelet Disord. 2017 Jan 10;18:10. doi: 10.1186/s12891-016-1365-4 (PMC5223391; doi:10.1186/s12891-016-1365-4)
Supplement: Additional file 4: — Association of multimorbidity of 2 or ≥3 diseases in- and excluding musculoskeletal disease with sick leave in the past 12 months in employed population ≤65 years, comparison of odds of sick leave between multimorbidity patterns including a musculoskeletal disease and multimorbidity patterns without a musculoskeletal disease. (DOCX 14 kb) [file 12891_2016_1365_MOESM4_ESM.docx]

| **Additional file** **4**  **Association of multimorbidity of 2 or ≥3 diseases in- and excluding musculoskeletal disease   with sick leave in the past 12 months in employed population ≤65 years** | |
| --- | --- |
|  | **OR [95%CI]** |
| **Single morbidity/Multimorbidity in- and excluding MSKD^*^** | |
| Musculoskeletal disease | 2.50 [2.01; 3.10] |
| Any disease excl. MSKD | 1.81 [1.55; 2.12]^a^ |
| 2 diseases incl. MSKD | 3.48 [2.51; 4.83] |
| 2 diseases excl. MSKD | 2.09 [1.51; 2.90] |
| ≥3 diseases incl. MSKD | 5.10 [3.15; 8.24] |
| ≥3 diseases excl. MSKD | 3.71 [1.80; 7.64] |
| *Results of multivariable logistic regression models adjusted for age, gender, level of education, origin, BMI and smoking status (n=5340)*  **reference category= healthy population*  *^a^ Significantly different from estimate for any disease excluding MSKD ^b^ Significantly different from estimate for multimorbidity of 2 diseases excluding MSKD ^c^ Significantly different from estimate for multimorbidity of* ≥3 *diseases excluding MSKD* | |
