# Supplementary material for: Work outcome in persons with musculoskeletal diseases: comparison with other chronic diseases & the role of musculoskeletal diseases in multimorbidity
Source: BMC Musculoskelet Disord. 2017 Jan 10;18:10. doi: 10.1186/s12891-016-1365-4 (PMC5223391; doi:10.1186/s12891-016-1365-4)
Supplement: Additional file 5: — Characteristics of the study sample and general Dutch population (DOCX 16 kb) [file 12891_2016_1365_MOESM5_ESM.docx]

| **Additional file 5 Characteristics of the study sample and general Dutch population** | | |
| --- | --- | --- |
| **Characteristics** | **Study population** | **General Dutch population** |
| Age: years; mean (SD) [range] | 45.80 (11.0) [18 -65] | 48.31 |
| **Gender** | **n (%)** |  |
| women | 3008 (55.8) | 50% |
| **Education** | **n (%)** |  |
| primary school or no  education | 105 (2.0) | 30.49% |
| lower professional education | 544 (10.2) |  |
| middle or secondary professional   education | 1677 (31.3) | 21.59% |
| secondary education | 567 (10.6) | 38.11% |
| university education | 2464 (46.0) |  |
| **Origin** | **n (%)** |  |
| western | 8332       (94.70) | 89% |
| 1^st^ generation non- western | 251         (2.85) | n/a |
| 2^nd^ generation non- western | 215         (2.44) | n/a |
| **BMI** | **n (%)** |  |
| underweight (<18.5) | 69 (1.3) | 1.65% |
| normal (18.5-24.99) | 2853 (52.9) | 49.37% |
| overweight (25.0- 29.99) | 1784 (33.1) | 36.76% |
| obese (≥30) | 690 (12.8) | 12.22% |
| *Reference:* CBS. Nederlanders naar opleidingsrichting, opleidingsniveau, leeftijd en geslacht. 2008. | | |
